# Development and Implementation of the AIDA International Registry for Patients With Undifferentiated Systemic AutoInflammatory Diseases

**DOI:** 10.3389/fmed.2022.908501

**Published:** 2022-06-10

**Authors:** Francesca Della Casa, Antonio Vitale, Giuseppe Lopalco, Piero Ruscitti, Francesco Ciccia, Giacomo Emmi, Marco Cattalini, Ewa Wiesik-Szewczyk, Maria Cristina Maggio, Benson Ogunjimi, Petros P. Sfikakis, Abdurrahman Tufan, Sulaiman M. Al-Mayouf, Emanuela Del Giudice, Emma Aragona, Francesco La Torre, Jurgen Sota, Sergio Colella, Ilenia Di Cola, Daniela Iacono, Irene Mattioli, Karina Jahnz-Rózyk, Rik Joos, Katerina Laskari, Carla Gaggiano, Anna Abbruzzese, Paola Cipriani, Gelsomina Rozza, Alhanouf AlSaleem, Derya Yildirim, Maria Tarsia, Gaafar Ragab, Francesca Ricci, Fabio Cardinale, Marcelina Korzeniowska, Micol Frassi, Valeria Caggiano, Moustafa Ali Saad, Rosa Maria Pereira, Virginia Berlengiero, Stefano Gentileschi, Silvana Guerriero, Teresa Giani, Viviana Gelardi, Florenzo Iannone, Henrique Ayres Mayrink Giardini, Ibrahim A. Almaghlouth, Riza Can Kardas, Djouher Ait-Idir, Bruno Frediani, Alberto Balistreri, Claudia Fabiani, Donato Rigante, Luca Cantarini

**Affiliations:** ^1^Department of Translational Medical Sciences, Section of Clinical Immunology, University of Naples Federico II, Naples, Italy; ^2^Department of Medical Sciences, Research Center of Systemic Autoinflammatory Diseases and Behçet's Disease Clinic, Surgery and Neurosciences, University of Siena, Siena, Italy; ^3^Rheumatology Unit, Department of Emergency and Organ Transplantation, University of Bari, Bari, Italy; ^4^Rheumatology Unit, Department of Biotechnological and Applied Clinical Sciences, University of L'Aquila, L'Aquila, Italy; ^5^Department of Precision Medicine, Università Degli Studi Della Campania Luigi Vanvitelli, Napoli, Italy; ^6^Department of Experimental and Clinical Medicine, University of Florence, Florence, Italy; ^7^Pediatric Clinic, University of Brescia and Spedali Civili di Brescia, Brescia, Italy; ^8^Department of Internal Medicine, Pulmonology, Allergy and Clinical Immunology, Central Clinical Hospital of the Ministry of National Defence, Military Institute of Medicine, Warsaw, Poland; ^9^University Department PROMISE “G. D'Alessandro”, University of Palermo, Palermo, Italy; ^10^Department of Pediatrics, Antwerp University Hospital, Edegem, Belgium; ^11^Center for Health Economics Research and Modeling Infectious Diseases (CHERMID), Vaccine & Infectious Diseases Institute (VAXINFECTIO), University of Antwerp, Antwerp, Belgium; ^12^Department of Rheumatology, Ziekenhuis Netwerk Antwerpen, Berchem, Belgium; ^13^KidZ Health Castle, Universitair Ziekenhuis Brussel, Jette, Belgium; ^14^Joint Academic Rheumatology Program, National and Kapodistrian University of Athens Medical School, Athens, Greece; ^15^Division of Rheumatology, Department of Internal Medicine, Gazi University Faculty of Medicine, Ankara, Turkey; ^16^King Faisal Specialist Hospital and Research Center, Riyadh, Saudi Arabia; ^17^Department of Maternal Infantile and Urological Sciences, Sapienza University of Rome, Polo Pontino, Italy; ^18^Division of Gastroenterology, Ospedali Riuniti Villa Sofia-Vincenzo Cervello, Palermo, Italy; ^19^Department of Pediatrics, Pediatric Rheumatology Center, Ospedale “Giovanni XXIII”, AOU Consorziale Policlinico, Bari, Italy; ^20^Department of Pediatrics King Faisal Specialist Hospital and Research Center Riyadh, Riyadh, Saudi Arabia; ^21^Internal Medicine Department, Rheumatology and Clinical Immunology Unit, Faculty of Medicine, Cairo University, Giza, Egypt; ^22^Faculty of Medicine, Newgiza University (NGU), Giza, Egypt; ^23^Rheumatology and Clinical Immunology, Spedali Civili and Department of Clinical and Experimental Sciences, University of Brescia, Brescia, Italy; ^24^Rheumatology Division, Hospital das Clinicas (HCFMUSP), Faculdade de Medicina, Universidade de São Paulo, São Paulo, Brazil; ^25^Unit of Rheumatology, Azienda Ospedaliero-Universitaria Senese, Siena, Italy; ^26^Department of Ophthalmology and Otolaryngology, University of Bari, Bari, Italy; ^27^Department of Clinical Sciences and Community Health, Research Center for Adult and Pediatric Rheumatic Diseases, ASST G. Pini-CTO, University of Milan, Milan, Italy; ^28^Rheumatology Unit, Department of Medicine, College of Medicine, King Saud University, Riyadh, Saudi Arabia; ^29^College of Medicine Research Center, College of Medicine, King Saud University, Riyadh, Saudi Arabia; ^30^Research Laboratory, Biodiversity, Biotechnology, Environment and Sustainable Development, Department of Biology, Faculty of Sciences, M'Hamed Bougara University, Boumerdes, Algeria; ^31^Bioengineering and Biomedical Data Science Lab, Department of Medical Biotechnologies, University of Siena, Siena, Italy; ^32^Ophthalmology Unit, Department of Medicine, Surgery and Neurosciences, University of Siena, Siena, Italy; ^33^Department of Life Sciences and Global Health, Fondazione Policlinico Universitario A. Gemelli IRCCS, Rome, Italy; ^34^Rare Diseases and Periodic Fevers Research Centre, Università Cattolica del Sacro Cuore, Rome, Italy

**Keywords:** autoinflammatory diseases, personalized medicine, precision medicine, rare diseases, International Registry

## Abstract

**Objective:**

This paper points out the design, development and deployment of the AutoInflammatory Disease Alliance (AIDA) International Registry dedicated to pediatric and adult patients affected by Undifferentiated Systemic AutoInflammatory Diseases (USAIDs).

**Methods:**

This is an electronic registry employed for real-world data collection about demographics, clinical, laboratory, instrumental and socioeconomic data of USAIDs patients. Data recruitment, based on the Research Electronic Data Capture (REDCap) tool, is designed to obtain standardized information for real-life research. The instrument is endowed with flexibility, and it could change over time according to the scientific acquisitions and potentially communicate with other similar tools; this platform ensures security, data quality and data governance.

**Results:**

The focus of the AIDA project is connecting physicians and researchers from all over the world to shed a new light on heterogeneous rare diseases. Since its birth, 110 centers from 23 countries and 4 continents have joined the AIDA project. Fifty-four centers have already obtained the approval from their local Ethics Committees. Currently, the platform counts 290 users (111 Principal Investigators, 179 Site Investigators, 2 Lead Investigators, and 2 data managers). The Registry is collecting baseline and follow-up data using 3,769 fields organized into 23 instruments, which include demographics, history, symptoms, trigger/risk factors, therapies, and healthcare information access for USAIDs patients.

**Conclusions:**

The development of the AIDA International Registry for USAIDs patients will facilitate the online collection of real standardized data, connecting a worldwide group of researchers: the Registry constitutes an international multicentre observational groundwork aimed at increasing the patient cohort of USAIDs in order to improve our knowledge of this peculiar cluster of autoinflammatory diseases. NCT 05200715 available at https://clinicaltrials.gov/.

## Introduction

Undifferentiated Systemic AutoInflammatory Diseases (USAIDs) represent a group of undefined medical conditions increasingly reported in medical literature. The acronym USAIDs identifies a subset of patients characterized by self-limiting episodes of inflammation that fail to meet criteria for the established monogenic or multifactorial autoinflammatory diseases, but display features of autoinflammatory disorders. Patients with USAIDs do not carry confirming pathogenic mutations in genes associated with monogenic autoinflammatory diseases and do not fulfill any of the diagnostic or classification criteria currently available for multifactorial autoinflammatory disorders. There are neither definite diagnostic criteria available, nor specific laboratory investigations for identifying USAIDs (1, 2). USAIDs can be suspected after ruling out infectious, neoplastic, autoimmune, and other monogenic and multifactorial autoinflammatory diseases. The concept of USAIDs has not been fully delineated neither in terms of diagnosis, nor in terms of optimal therapeutic approach, while long-term clinical evolution and prognosis have not been established at all. In this regard, the AutoInflammatory Diseases Alliance (AIDA) project is aimed at shedding new light on these conditions, potentially allowing their better definition and classification, and aimed at improving overall knowledge. To this aim, the creation of an International Network of expert physicians in autoinflammatory disorders combining together scientific efforts and sharing ideas, information, and the development of an International Registry to collect data from dedicated centers around the world would be precious.

Priority of the AIDA project is the development and maintenance of international registries for patients affected by monogenic and multifactorial autoinflammatory disorders and ocular inflammatory diseases. To date, nine international registries have been launched, including Behçet's disease (BD), monogenic autoinflammatory diseases, Still's disease, Schnitzler's syndrome, Periodic Fever, Aphthous stomatitis, Pharyngitis and cervical Adenitis (or PFAPA) syndrome, non-infectious uveitis, non-infectious scleritis, vacuoles, E1 enzyme/X-linked autoinflammatory somatic (or VEXAS) syndrome and undifferentiated autoinflammatory diseases. The use of these registries will allow sharing of knowledge, experience, and different perceptions on the clinical, therapeutic and research approaches in the field of rare diseases (3, 4).

This paper purposes to point out the design, development and deployment of the AIDA International Registry dedicated to patients with USAIDs, which corresponds to a multicentre, non-interventional, population- and electronic-based observational clinical study. The registry will include patients with unexplained systemic inflammation suggested by laboratory, clinical or therapeutic clues which will be widely clarified in the methods section.

## Materials and Methods

### Study Design

The AIDA Registry for USAIDs patients has been conceived to collect both retrospective and prospective data. In particular, retrospective phase refers to demographic, clinical, laboratory, instrumental and therapeutic information available at the time of enrollment into the Registry; prospective phase includes clinical, therapeutic and socioeconomic data acquired thereafter.

Variables included in the Registry have been selected on the basis of clinical and laboratory features generally described in patients with autoinflammatory diseases; evidence currently available in literature; therapeutic details required to comprehensively describe treatment options proposed for monogenic and multifactorial autoinflammatory diseases, and information required during the follow-up visits according with the best standard of care.

Some information may be repeated and, therefore, recorded in both the retrospective and prospective phases, as for example data about the cardiovascular risk and information about fertility, pregnancy and breastfeeding period. The retrospective assessment includes clinical and laboratory data referring to the start of symptoms, the time at the diagnosis, and the time at the enrollment into the Registry; for each treatment performed during the patient's history, clinical and laboratory data would be required referring to the start of the treatment, the 3-, 6- and 12-month visits and at the last assessment while on the treatment. Conversely, the follow-up visits will be added at the patient re-evaluations following the inclusion in the AIDA Registry; a follow-up re-evaluation should take place at least every year and at any change in the treatment strategy, as for the introduction of new drugs and posology changes.

Since only data related to the standard routine management are recorded and no additional specific investigations are required, no funds are provided for patient's enrollment and no further impact on national healthcare will be determined by the participation in the AIDA project. Similarly, treatments administered prior or after the enrollment in the AIDA Registry are drawn by the best standard of care and are not influenced by the study protocol.

Any center managing USAIDs may participate in the project without limitations regarding the location, medical specialty, or type of practice setting. The centers that would like to participate, can make a request by contacting the Promoter directly or by sending an email from the web page contact the AIDA Team by writing to support@aidaregistry.org or using a specific form at the bottom of the following page: https://aidanetwork.org/en/aida.

The only required prerequisite is obtaining approval from the local Ethics Committee and appointing a Principal Investigator whose function is coordinating the study locally, and Site Investigators responsible for the documentation and data entry for that site.

### Registry Objectives

Among the objectives of the USAIDs Registry, the primary one is to enroll the largest number of patients with systemic inflammation that potentially involves all organs and tissues due to dysfunction of the innate immunity, which cannot be framed in the field of monogenic or multifactorial autoinflammatory diseases. At current, criteria for enrolling patients in the basket of USAIDs include: (a) presence of recurrent stereotyped clinical manifestations with no symptoms between episodes; (b) positive family history despite lack of genetic mutations; (c) hematological disorders associated with somatic mutations (e.g., *RUNX1, BCOR, WTI* or *TP53* genes); (d) increased inflammatory markers during attacks (serum amyloid-A, erythrocyte sedimentation rate, C-reactive protein); (e) laboratory-proved evidence of *NLRP3* inflammasome activation; (f) increased serum levels of interleukin (IL)-1 and/or IL-6 during clinical manifestations; (g) effectiveness of colchicine or corticosteroid administration; (h) response to IL-1 or IL-6 inhibitors; (i) absence of cyclic neutropenia, immunodeficiency, chronic infections, inflammatory bowel diseases, autoimmune diseases or neoplasms explaining the systemic inflammatory picture.

Further objectives include: (a) the identification of new genetic syndromes in addition to those currently included among monogenic autoinflammatory diseases; (b) the categorization into subgroups characterized by similar signs and symptoms and response to therapies according to a_clustering method; (c) the search for new diagnostic or classification criteria; (d) the assessment of disease complications and life-threatening sequelae; (e) the identification of predisposing factors for a worse outcome and systemic amyloidosis development; (f) the detection of biomarkers and predictive factors for disease monitoring; (g) the identification of variables capable of identifying patients more likely responsive to different therapeutic approaches; (h) the description of the socioeconomic impact of these diseases in association with the epidemiologic burden in different geographic contexts; (i) the evaluation of current clinimetric tools in different diseases and contexts alongside with assessment of new clinimetric instruments specific for USAIDs patients; (j) the behavior of disease during pregnancy and post-partum period; (k) the impact of chronic disease inflammation on the cardiovascular risk. Table 1 summarizes all objectives of USAIDs Registry.

**Table 1:** Objectives of the USAIDs registry.

Main objective	Built an International Registry to overcome limitation of small number of USAIDs patients in each single center
Other objectives	Identification of new genetic syndromes
	Categorization into subgroups characterized by similar signs and symptoms and response to therapies
	Search for new diagnostic or classification criteria
	Assessment of disease complications and life-threatening sequelae
	Identification of predisposing factors to a worse outcome and systemic amyloidosis development
	Detection of biomarkers and predictive factors for disease monitoring
	Identification of variables capable of identifying patients more likely responsive to different therapeutic approaches
	Description of socioeconomic impact of these diseases
	Assessment of new clinimetric instruments specific for USAIDs patients
	Behavior of disease during pregnancy and post-partum period
	Impact of chronic disease inflammation on the cardiovascular risk

When a substantially high number of patients is enrolled, more specific and more cutting-edge studies will be proposed in the context of the AIDA network.

### Inclusion/Exclusion Criteria

Primary inclusion criterion is the presence of systemic inflammation driven by innate immunity (5) with the identification of clinical features resembling other autoinflammatory diseases. Furthermore, as USAIDs are based on an exclusion diagnostic approach (1), patients with monogenic and multifactorial autoinflammatory diseases, alongside patients with infections, malignancies or autoimmune diseases, are excluded from the Registry. Multifactorial autoinflammatory diseases will have to be ruled out when the diagnostic/classification criteria are not fulfilled for Behçet's disease (6, 7) Still's disease (8, 9), PFAPA syndrome (10, 11), Schnitzler's disease (12), and Chronic Recurrent Multifocal Osteomyelitis (13).

Infectious, neoplastic and autoimmune diseases have to be firstly excluded according to the best standard of care for any of those clinical conditions.

Patients included in the USAIDs Registry should have been preliminarily assessed in order to exclude monogenic autoinflammatory diseases through a Next Generation Sequencing (NGS) approach, when available, or using other methods of genetic sequencing based on the specific patient's clinical framework. In addition, patients fulfilling clinical diagnostic criteria for Familiar Mediterranean Fever (14–16), have to be excluded.

### Ethics

The first national regulatory approval of the AIDA project has been obtained in June 2019 by the Ethics Committee of Azienda Ospedaliera Universitaria Senese, Siena, Italy (Ref. N. 14951). Later, expert centers for the diagnosis, clinical management and treatment of autoinflammatory diseases have approved the project across Europe, the Middle East, North Africa and the North America and the South America and actively participate in the AIDA International Registry.

This project, already registered at ClinicalTrials.gov (ID: NCT05200715), follows Declaration of Helsinki recommendations. All patients enrolled have to provide their written informed consent after having been carefully informed about the project and its aims, long-term purposes, lack of any impact on their clinical and therapeutic course. The possibility to refuse entering or withdrawing from the study at any time with no impact on the clinical management is well-cited and patients have to be informed about the personal data privacy and security in accordance with the local and/or European regulations. As far as adolescents are concerned, their parents (or legal representatives) have to comply with the study requirements during the whole study.

Both patients and Principal Investigators may withdraw their consent to use of data for statistical analyses at any time. If a patient withdraws the consent, no further data for that patient will be entered into the Registry and, if requested by the patient, all prior data will be deleted soon after her/his request to the Promoter. Patients' data are collected and stored in accordance with the EU General Data Protection Regulations (GDPR) on the processing of personal data and protection of privacy (2016/679/EU) (17).”

### Online Data Collection and Management

Data are collected through Research Electronic Data Capture (REDCap), which is an electronic data capture tool developed at the Vanderbilt University Medical Center. It is hosted at Virginia Commonwealth University (Award Number UL1TR002649) and can be also used to develop patients' registries. The software is distributed at no costs and currently about over 5,700 Institutions in 145 Countries have already joined this online opportunity (18).

Investigators included into the AIDA project can log in the Registry through the REDCap web-interface and later insert data on the Instruments (pages) of the Registry. None of the recruited Principal Investigators and Site Investigators are allowed to see information inserted by other Centers. The electronic data entry system of the Registry is in English.

While public website of the AIDA Network (https://aidanetwork.org/en/) may be accessed by anyone who wants to learn about this Project, the Registry website (https://sitbio.med.unisi.it/redcap/redcap_v12.2.1/index.php?pid=40) is hosted separately and requires credential to meet data privacy regulations.

The Investigators will be responsible for entering the own study data in the online Registry. They will be also responsible for the accuracy of the information accrued, with the Principal Investigator required to supervise the accuracy of the data. The security of the patients' information is guaranteed by the online access through personal username and password and by the compliance of the Investigators with local legislation. Each Principal Investigator and Site Investigator may provide their study proposal during dedicated meetings.

**Figure 1:**
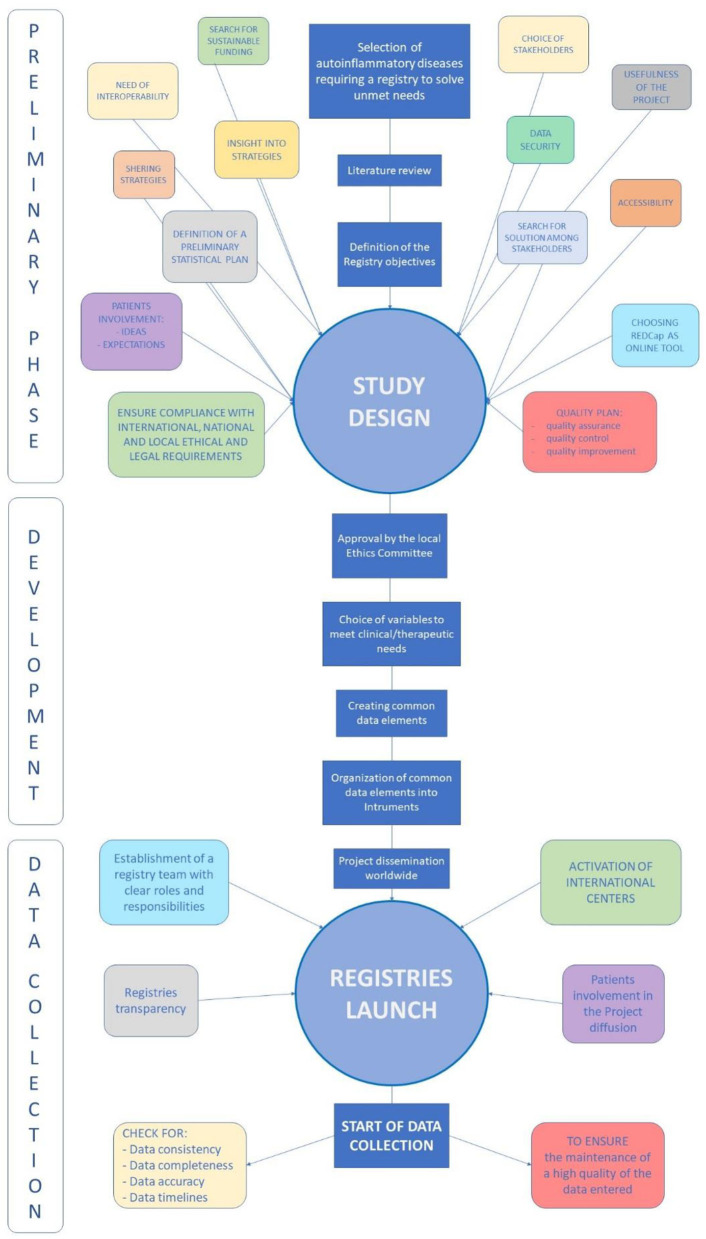
Summary of the main phases corresponding to the creation of the Registry: the preliminary phase with all the elements required to carry out the project; the development phase during which common data elements and instruments of the Registry were realized; the data collection phase following the launch of the Registry accompanied by the efforts to maintain the high quality of data entered and of the Registry according to the new scientific acquisition.

Figure 1 provides a summary of the three main phases describing the creation of the Registry, pointing out all the elements contributing to development of the project, realization of the online tool based on REDCap, and launch of the Registry followed by data collection.

### Statistical Analysis

Statistical analysis will include, in addition to descriptive statistics, also correlations between groups and comparisons among subgroups; furthermore, machine learning principles will be applied to complement the conventional data analysis. Other statistical analysis will depend on the objectives to be achieved over time and on the type and number of data collected.

Each Principal Investigator may analyze data collected in the own center, does conducting prospective and retrospective studies of the behalf of the AIDA Network; the totality of data collected in the Registry will be managed by statistics and physicians involved in the network, selected by the Promoter on a case-by-case basis according with their field of expertise. All the variables related to the study's objectives will be provided to the Investigators who will take care of the study. Analysis of data will take place according with the aims of the study, scientific relevance, biologic plausibility, and the number of data collected.

## Results

**Figure 2:**
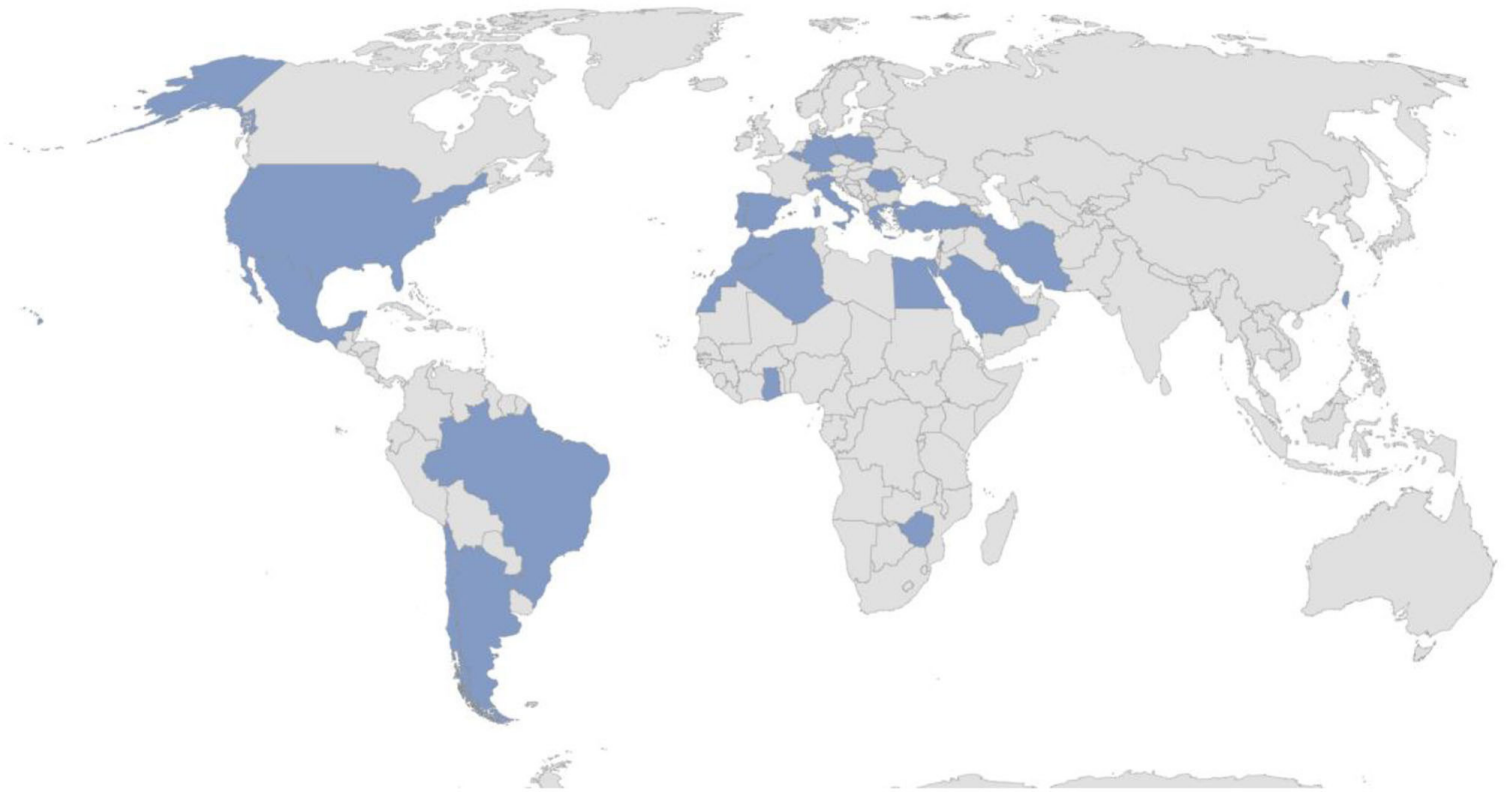
Country involved in the AIDA Network (update February 7^th^, 2022).

This International Registry was created with the essential purpose to obtain solid scientific information about rare autoinflammatory disorders not thoroughly studied yet. Indeed, this project may quickly reach a wide geographic coverage, as shown during the last 20 months. In particular, Centers from 23 Countries (Algeria, Argentina, Belgium, Brazil, Chile, Egypt, Germany, Ghana, Greece, Iran, Italy, Lebanon, Mexico, Morocco, Poland, Portugal, Romania, Saudi Arabia, Spain, Taiwan, Turkey, United States, Zimbabwe) in 4 Continents have already joined the Project sharing their knowledge and experience about autoinflammatory diseases. At current (February 7^th^, 2022), 110 centers around the world corresponding to 290 users (111 Principal Investigators, 175 Site Investigators, 2 Lead Investigators, 2 data managers) have already joined the network (Figure 2).

### Registry Development

In order to better record patients' clinical and therapeutic history, a wide number of variables has been included into the Registry to describe in detail the whole disease course; general and specific items have been included to shed light on a still unknown clinical entity. Therefore, a total number of 3,357 common data elements (fields corresponding to variables) have been created and organized into 23 instruments (forms corresponding to different pages) to constitute the USAIDs Registry.

The common data elements refer to patient's demographics, medical history, laboratory features, genetic characteristics, comorbidities, symptoms at disease onset, symptoms developed over time, cardiovascular risk, work-up examinations, pregnancies after symptoms onset, disease complications, long-term clinical outcomes, treatments administered, short- and long-term responses to treatments, management of different therapeutic strategies in terms of posology changes and drug combinations, and impact on national healthcare. Specific fields appear only if required based on patient's clinical history, thanks to a branching system of questions. Therefore, only a small part of the 3,769 fields appears to the Investigators for each patient, and the number of questions to be answered depends exclusively on the complexity of the patient's clinical history. With regard to the prospective phase of the AIDA project, longitudinal data are acquired using a specific follow-up instrument, which includes details about clinical and laboratory features and treatments update.

Data elements from AIDA Registries for other autoinflammatory diseases are shared, while specific data elements for USAIDs have been added to describe the specific field of these entities.

Furthermore, a specific instrument includes items possibly setting up an early form of clinical diagnostic scores and criteria; this instrument describes the reasons leading to the enrolment of the patient among USAIDs. In addition, the Eurofever scores and diagnostic/classification criteria for other multifactorial autoinflammatory diseases have been included in this instrument (10, 11, 14, 15, 19).

The Instruments constituting the Registry and their reference time-points are listed in Table 2.

**Table 2:** List of instruments (forms) included in the AIDA International Registry dedicated to USAIDs patients, with the corresponding number of common data elements, time-points at which they should refer to and number of mandatory fields included.

**Instruments**	**Fields**	**Retrospective/prospective phase**	**N. of mandatory fields**
Demographics	10	Retrospective phase	4
Consents	4	Retrospective phase	2
Diagnostic data and family history	24	Retrospective phase	2
General genetic information	5	Retrospective phase	1
Gene mutations	7	Retrospective phase	0
Features of attacks during childhood	42	Retrospective phase	0
Features of attacks at disease onset	43	Retrospective phase	0
Features of attacks up to the diagnosis	56	Retrospective phase	0
Features of attacks up from diagnosis to the time of enrolment in the AIDA Registry	56	Retrospective phase	0
Clinical diagnostic scores and criteria	10	Retrospective phase	0
Laboratory data	17	Retrospective/prospective phase	1
Cardiovascular risk	24	Retrospective/prospective phase	2
Past and current treatments	1	Retrospective phase	0
NSAIDs monotherapy-the retrospective phase	33	Retrospective phase	1
Corticosteroid monotherapy/main therapy-the retrospective phase	102	Retrospective phase	1
Colchicine treatment-the retrospective phase	50	Retrospective phase	1
*Streptococcus salivarius* K12 treatment-the retrospective phase	54	Retrospective phase	1
Treatment with cDMARDs (not associated to biotechnological agents)-the retrospective phase	315	Retrospective phase	6
Treatment with small molecules (not associated to biotechnological agents)-the retrospective phase	606	Retrospective phase	12
Treatment with biotechnological agents-the retrospective phase	1,029	Retrospective phase	14
Fertility and pregnancy	14	Retrospective/prospective phase	1
Disease course and treatment during pregnancies	66	Retrospective/prospective phase	1
Follow-up visits: clinical manifestations and treatment-the prospective phase	766	Prospective phase	58

### Patients' Involvement

In the last few years, the role of patients has progressively been increasing to become central in stimulating the research effort and quality of clinical management (20); furthermore, the role of patient advocacy organizations has added an important new dynamic to the patient care (21). Currently, patient advocacy groups may help in many ways, by disseminating information, supporting the recruitment of patients, and taking part in regulatory processes. There are different associations actively involved in the AIDA project, including the Italian Association of Periodic Fever (A.I.F.P., *Associazione Italiana Febbri Periodiche*), the National Association of People with Rheumatic and Rare Diseases (A.P.M.A.R., *Associazione Nazionale Persone con Malattie Reumatologiche e Rare*) and the National Association of Rheumatic Patients (A.N.M.A.R., *Associazione Nazionale Malati Reumatici*). More in detail, the associations of patients were asked to provide their opinion since the very beginning of the AIDA project; patients' point of view and impressions were required before starting the project, while concerns, especially regarding the protection of personal data, were carefully answered. Patients did not desire a registry mainly focused on therapeutic aspects; in fact, we expanded the focus to all the different issues of the disease. To date patient organizations are of Italian origin only; however, other international centers have been invited to facilitate the participation of other organizations worldwide by diffusing the project among patients.

## Discussion

In the last years, the use of online networks has gradually increased, facilitating the spreading of knowledge and establishing worldwide research collaboration between researchers, clinicians, pharmaceutical companies, patients' advocatory groups, and patients with their families. This constitutes a great revolution in the field of rare diseases, where the small number of cases and the difficult-to-reach diagnosis are barriers to translational research and the identification of a large patient cohorts. In this context, the AIDA network and the corresponding registries dedicated to rare diseases have been created as a unifying agent in the field of autoinflammatory diseases to gather forces currently spread across the various referring centers. This highly concerns USAIDs, which correspond to a cluster of medical conditions lacking a widely shared classification and definition along with an internationally accepted protocol for management and treatment. Indeed, the concept of USAIDs itself is at an early stage of development and the knowledge on this clinical group of diseases is embryonic at current.

The AIDA network has been also created to facilitate the international consultation and involvement of all medical figures dealing with the management of autoinflammatory diseases in general, and USAIDs in particular. Actually, the AIDA network already includes and enhances the communication between different specialties, such as rheumatologists, immunologists, gastroenterologists, dermatologists, ophthalmologists, internal medicine physicians, geneticists and pediatric rheumatologists for patients with childhood onset disease. This project represents a demonstration of how well a web-based worldwide collaboration can overcome the fragmentation of clinical and research experiences, expanding and improving our knowledge in the field of rare and complex diseases.

The AIDA project has met the expectations for the development of an online platform dedicated to patients with autoinflammatory diseases, including those characterized by a clear but undifferentiated clinical picture. This represents the first fundamental step to overcome the limitations related to the poor number of patients included in case-series and small individual studies currently available (22). This is even more remarkable when considering that current research aspires to a personalized medicine aimed at choosing the most proper treatment according to the baseline features and risk of developing severe manifestations or complications. In this light, a large-scale, long-term patient's Registry is essential to provide additional evidence on still unknown autoinflammatory diseases included in the USAID acronym. In this regard, a wide number of patients is required to assess atypical and borderline cases especially when aiming at identifying common clinical features that cluster in a specific new nosologic entity. The identification of new genetic syndromes and/or diagnostic/classification criteria is one of the most ambitious goals of this Registry. A further goal is to better characterize specific phenotypes of patients, as for musculoskeletal and abdominal pain, which have been frequently described in USAIDs (2, 23).

Though an International Registry enables the recruitment of a high number of patients that is required to assess response to treatment and prognostic variables among specific subgroups of patients. A long-term observational Registry could allow assessing any change in the natural history of USAIDs as a consequence of the new therapeutic approaches available at current. Furthermore, the efficacy of a therapeutic strategy might suggest the involvement of a particular molecular pathway in disease pathogenesis of specific patient subgroups.

The Registry may also be a precious source of real-life data regarding the role of chronic inflammation on the cardiovascular risk as well as disease behavior during pregnancy and postpartum. The prospective phase will also provide data about the socioeconomic impact of these diseases and benefits that national healthcare could obtain from different therapeutic strategies.

As a whole, achieving all the goals aspired by this Registry will improve the knowledge about this peculiar basket of inflammatory disorders and facilitate their early diagnosis, with a consequent decrease of long-term or life-threatening complications, such as amyloidosis, and a positive impact on quality of life and life expectations (2). Noteworthy, the Registry is flexible to include other future unmet needs and implement protocol variations according with future clues and suggestions deriving from future scientific progress. Indeed, this Registry could potentially communicate with other existing or future similar instruments.

The AIDA Registry for USAIDs patients has the usual shortcomings typically present in observational studies, especially selection biases deriving from the number of missing data and any non-consecutive enrolment of patients. In addition, entering data into the Registry requires time and attention, especially when patient's medical history is particularly complex, as for patients with long-term disease course, multiple treatment approaches attempted over time and many posology changes to report. Nevertheless, entering retrospective data requires 2–3 h, while completing the form at follow-up visits takes a maximum of 10–15 min. Despite its limitations, this Registry has the potential, given also its geographical extension, to eventually achieve all the objectives thus shedding light on a quite unknown field of autoinflammation.

Of note, a branch of the AIDA project defined as “AIDA for patients” is under development. “AIDA for patients” is an online tool based on the REDCap technology primarily aim at involving patients in the collection of the data, especially regarding the impact of the disease on the quality of life and on socio-economic aspects and the current status of the disease with specifically built patients reporting outcome (PROs). Moreover, “AIDA for patients” was born to enhance patients' participation in the decision-making process when establishing the lines of research and the strategies to follow, in order to seek the growth of the AIDA project.

In conclusion, the AIDA International Registry for USAIDs patients has been developed and activated to facilitate the collection of standardized data and enable international multicentre collaborative research. Data sharing, implementation, and optimisation of research about autoinflammatory diseases, along with international consultation, and dissemination of knowledge represent pivotal goals that may be easier to achieve via this international effort offered by the AIDA network.

## Ethics Statement

The studies involving human participants were reviewed and approved by Azienda Ospedaliera Universitaria Senese, Siena, Italy (Ref. No. 14951). Written informed consent to participate in this study was provided by the participants' legal guardian/next of kin.

## Author Contributions

FD wrote the first draft of the manuscript. AV conceived and designed the study and revised the draft of the manuscript. DR revised the draft of the manuscript. LC conceived and designed the study and accounts for AIDA Registries Coordinator. AB is the bioengineer involved in the technical management of the platform and registries. GL, PR, FCi, GE, MC, EW-S, MM, BO, PS, AT, SA-M, ED, EA, FL, JS, SC, ID, DI, IM, KJ-R, RJ, KL, CG, AAb, PC, GRo, and AAl were involved in data recruitment in the Registry dedicated to patients with USAIDs. DY, MT, GRa, FR, FCa, MK, MF, VC, MS, RP, VB, SGe, SGu, TG, VG, FI, HG, IA, RK, DA-I, BF, and CF included in the authorship as investigators from the top contributor centers for any of the other seven AIDA Registries. Authorship has been established based on the number of data recruited in the AIDA Registries on February 7th, 2022. All authors contributed to the article and approved the submitted version.

## Conflict of Interest

The authors declare that the research was conducted in the absence of any commercial or financial relationships that could be construed as a potential conflict of interest.

## Publisher's Note

All claims expressed in this article are solely those of the authors and do not necessarily represent those of their affiliated organizations, or those of the publisher, the editors and the reviewers. Any product that may be evaluated in this article, or claim that may be made by its manufacturer, is not guaranteed or endorsed by the publisher.
